# Nasty wars and needy veterans? How cognitive polyphasia may explain conceptualizations of the U.S. Iraq and Afghanistan veterans as victims and heroes

**DOI:** 10.3389/fsoc.2024.1442649

**Published:** 2024-08-15

**Authors:** Rita Helena Phillips

**Affiliations:** Department of Education, University of Klagenfurt, Klagenfurt, Austria

**Keywords:** civil–military relations, Iraq, Afghanistan, reintegration of veterans, transition

## Abstract

Representative opinion polls indicate that members of the U.S. public may hold dichotomous perceptions of their veterans. While the majority of the U.S. public appreciates and honors their veterans, they are also considered to suffer from war-induced trauma and physical disabilities. Victimizing attitudes toward the veteran population may result in stigmatization and a more difficult transition into civilian society. This may be particularly problematic for U.S. veterans who were deployed to Iraq and Afghanistan as this younger veteran population needs to reintegrate not only into civilian society but also into civilian workplace settings. The present study aims to uncover and unravel underlying rationalities that justify heroizing and victimizing sentiments in relation to Iraq and Afghanistan veterans. In order to delve beyond socially desirable reporting and cultural norms, in-depth semi-structured interviews with 29 individuals (20 non-veterans and 9 veterans) were conducted. Three themes were identified by thematic analysis: Theme 1 “Individual Understandings of the Deployments to Iraq and Afghanistan” represents an underlying framework that tainted perceptions of Theme 2 “Conceptualizations of war, deployment, and violence” and Theme 3 “Evaluations of the veteran’s personality.” If the deployments were considered justified, then veterans were heroized, characterized with supreme altruistic traits when compared with civilians. Negative effects on health that were arbitrarily related to deployment experience were classified as short-lived. If the deployments were scrutinized, then veterans were considered as naïve victims of a deceitful government, suffering from long-term health problems. Importantly, as discussions surrounding the legitimacy of the deployments were context-dependent, the participants were able to hold perceptions of veterans as victims and as heroes side by side. In conclusion, the heroization and victimization of veterans may be the result of considering different viewpoints, elucidating diversity and access to equivocal information in an increasingly complex social world. Although the present findings may require further validation, they suggest that changing negative, stereotyping perceptions of veterans may require a coherent rationale for deployments and uniform mission objectives.

## 1 Introduction

Research has produced an extensive series of compelling evidence that suggests veterans can be commonly associated with heroizing and victimizing sentiments across different cultures and societies (Coy et al., 2008; Gibson and Condor, 2009; Gibson, 2012; Phillips et al., 2022; Phillips and Albanesi, 2022; Phillips et al., 2023). To date, limited attempts have been made to illuminate a possible relationship between victimizing and heroizing sentiments. The interaction between heroizing and victimizing sentiments may be particularly evident in perceptions of veterans from the most recent major U.S. deployments, Operation Enduring Freedom in Afghanistan (OEF), and Operation Iraqi Freedom/Operation New Dawn (OIF/OND) as these operations have been among the costliest and publicly most disputed U.S. deployments of the 21st century. With almost 3 million U.S. service personnel deployed to Afghanistan or Iraq, over 7,000 fatalities and more than 53,000 service personnel wounded in action (US Department of Defense, 2021), and public appreciation for these missions decreased significantly over time. While the public’s support for the U.S. missions was strong at the onset, with the majority of the U.S. public being in favor of these deployments (Gallup, 2001; Gallup, 2024), support waned significantly when U.S. troops continued to take on heavy casualties (Washington Post, 2011). Today, the majority of the U.S. public believes that the wars in Iraq and Afghanistan were not worthwhile fighting and that both missions were failures (Washington Post, 2011; Gallup, 2021; NPR, 2021; Pew Research Center, 2021).

Public dissent with the missions in Iraq and Afghanistan may have problematic consequences for veterans returning from these missions. While some research suggests that the U.S. public may be able to differentiate between their perception of the missions and the veterans who returned from these missions (Pew Research Center, 2011, 2018), it is not unreasonable to suspect that long running public scrutiny may also taint public perceptions of veterans negatively. Specifically, numerous governmental and independent commissions, finding no factual basis for the governmentally perpetuated mission aims (Doherty and Kiley, 2023), fueled growing public dissent regarding the missions, framing these as illegitimate wars (Connah, 2021; Akhtar, 2023). This is problematic as evidence suggests that if wars are perceived to be illegitimate, then veterans who returned from these wars are represented in the context of suffering from PTSD and trauma (McGarry, 2012). While socially appreciated missions (i.e., WW1 and WW2) may also draw attention to the veteran as suffering from experiencing atrocities, the social focus shifts slightly in the context of negatively perceived missions (c.f. Goldensohn, 2006). Cultural representations of veterans that reside in media, literature, and films then draw almost exclusively attention to horrific experiences on the battlefield (i.e., De Groot, 1995; Chattarji, 2000; Goldensohn, 2006). From this perspective, veterans require public sentiment, empathy, and pity as conceptualizations of the veteran overlap with definitions of victims in a victimological context (McGarry, 2012). In a victimological context, “civilians and soldiers in a war” (Kauzlarich et al., 2001, p. 175) can be considered to be victims of a “state crime.” Obliged by the military contract, the soldier is forced to violate international and/or domestic laws in addition to human rights standards (*ibid*). In this sense, “soldiers […] while ‘doing their unpleasant, ennobling duty’, are being victimized by the State and corporate actors” (Ruggiero, 2005, p. 251).

A popular example of the cultural victimization of veterans following unpopular wars is U.S. veterans who returned from Vietnam. The publicly perceived illegitimacy of the intervention based the image of the Vietnam veteran upon readily accessible social narratives of injustice and violence (Thomson, 1998). The returning Vietnam veteran became a representation of defeat and a victim of violence, comparable to a civilian Vietnamese (Wessely, 2005), troubled by war-induced psychological trauma. Here, scholars outline a shift in public perceptions of war-induced trauma (Wessely, 2005). While psychopathological conditions in WW1 and WW2 were considered either short-lived or long-term-lived, though attributed to other germane circumstances unrelated to war, Vietnam war veterans were perceived to suffer from long-lived psychological trauma directly related to combat experience (Wessely, 2005). The impact of their combat experience was considered to torment veterans, becoming volatile and isolated ‘others’ in U.S. society and continuing to fight illusionary battles as a result of their experiences (Wessely, 2005; McNally, 2006).

Problematically, research suggests that similar tendencies are evident in the public representation of U.S. Iraq and Afghanistan veterans. Evidence outlines that the majority of U.S. media may frame U.S. Iraq and Afghanistan veterans as suffering but deserving victims of war experience (Bragin, 2010; Kleykamp and Hipes, 2015; Rhidenour et al., 2019). This issue is further evidenced by studies outlining an increase in media reports about PTSD in soldiers during the Afghanistan and Iraq wars. Between the years 2003 and 2006, U.S. news media coverage of PTSD increased by 136% and to 211% in the subsequent 2 years culminated with the 2008 presidential election (Armstrong and Olatunji, 2009). Importantly, evidence, comparing the prevalence of PTSD in veterans from different wars, suggests that U.S. Iraq and Afghanistan veterans are in fact less likely to develop PTSD when compared to U.S. Gulf and Vietnam war veterans (National Center for PTSD, 2016). While some of this may be explained by higher survival rates and improved medical care for Iraq and Afghanistan veterans (Goldberg, 2016), it remains questionable why the media focused predominantly on representing veterans in victimized ways.

### 1.1 Ambivalent perceptions of veterans: victimizing and heroizing sentiments

Media framings of U.S. Iraq and Afghanistan veterans as a labile and damaged population are mirrored in U.S. public perceptions of veterans. For example, U.S. opinion polls and surveys with representative sample sizes suggest that a majority of the U.S. population may associate violent behavior and emotional instability with Iraq and Afghanistan veterans (Pew Research Center, 2011, 2019). In fact, public estimations regarding the prevalence of physical and psychological health problems in U.S. Iraq and Afghanistan veterans exceed actual health concerns by far (Hoge et al., 2006; Hipes et al., 2015; Schreger and Kimble, 2017). This is problematic as negative preconceptions of U.S. Iraq and Afghanistan veterans’ physical and mental health may have deleterious effects on the transition and reintegration of veterans into civilian society. It is well evidenced that mentally and physically ill individuals are publicly discriminated against and perceived to have diminished competence (*cf.* Link et al., 1999; Greene-Shortridge et al., 2007; Ben-Zeev et al., 2012; Hipes et al., 2015). As such, public beliefs about U.S. Iraq and Afghanistan veterans that relate veterans to suffering from ill health lead to economic disadvantages and implicit stereotyping. For example, studies demonstrated that stereotypical information about veterans was used by hiring managers to the potential detriment of veterans (Stone et al., 2018) and that labeling veterans with PTSD led to stereotypical attitudes toward veterans and negative preconceptions about this population by members of the public (Hipes and Gemoets, 2019). Particularly, Army veterans may be prone to stigmatizing beliefs as the Army is commonly associated with front-line experience and face-to-face fighting that compares to the culturally perpetuated ‘horrors of war’ of WW1 and WW2 front-line fighting (*cf.* Heffernan, 1995; Fussell, 2009; Woodward and Jenkings, 2013). As research finds people relate mental disability particularly to combat experience (MacLean and Kleykamp, 2014), it may stand to reason that Army veterans are the most prone branch of service to be associated with mental ill health. Moreover, the investigation of U.S. Army Iraq and Afghanistan veterans had practical considerations. As a younger veteran population, veterans from the most recent missions may be most disadvantaged by erroneous stereotypes. For example, erroneous attributions of mental and physical ill health may affect the veteran’s economic situation and thus hinder a successful transition to civilian life.

In addition, previous research suggests that veterans who perceive a lack of respect and pride for homecoming are more likely to have problems with the adaption to civilian life and develop PTSD and suicidal thoughts (e.g., Butler et al., 1988; Solomon et al., 1990; Fontana and Rosenheck, 1994; Bolton et al., 2002; Boscarino et al., 2018). In fact, homecoming support was a stronger predictor of PTSD and suicidal thoughts than theatre or combat exposure itself (Johnson et al., 1997; Boscarino et al., 2018). Similarly, recent evidence suggests that public regard for veterans may impact depression, self-efficacy, and life satisfaction. If veterans perceive the public to appreciate them, they are less likely to experience depression and score higher on self-efficacy and life satisfaction scales.

Despite these widespread public health concerns, polls and surveys outline that the majority of the U.S. public may also hold positive beliefs about U.S. Iraq and Afghanistan veterans, characterizing veterans as a dedicated, competent, and capable population with a lot to offer (Pew Research Center, 2019; Veterans and Citizens Initiative, 2022; Wenig et al., 2023). Although the majority of the U.S. public is ambivalent about the value of the veterans’ efforts in Iraq and Afghanistan, they respect and appreciate their veterans (Pew Research Center, 2018). This may be rooted in U.S. Military culture, in which service personnel and veterans are routinely celebrated as heroes in public spheres. While veterans may be perceived to be victims of war, possibly even as a physical representation of the costs of war, they are still held in high regard. However, high proportions of the U.S. population believing that “Iraq and Afghanistan veterans have served honorably, but given their stress and sacrifice, they should be allowed to recover and other people should lead” (Greenberg Quinlan Rosner Research, 2012) are still problematic, as stigmatizing veterans. As such veterans may be appreciated and honored for their service, while also being victimized.

In conclusion, U.S. Iraq and Afghanistan veterans may be ambivalently viewed by the public. They are perceived as strong and capable leaders who can be valuable assets for their country, yet struggling with health problems and mental lability. However, while public perceptions of veterans and the impact of public perceptions on the veterans’ transition into civilian society have been well documented, little is known about the underlying reasons why individuals may hold their perceptions of veterans. Here, particularly, the relationship between heroic and victimized understandings of veterans has been neglected by previous research. The limited previous research that has been conducted in this field almost exclusively examined how sociodemographic characteristics may impact the extent to which individuals may hold victimizing sentiments toward veterans by drawing on quantitative or mixed methodologies (Phillips et al., 2022; Phillips and Albanesi, 2022; Phillips et al., 2023). As research has not yet examined relationships between heroic and victimizing sentiments, the present study addressed this gap by taking a qualitative approach by drawing on social representation theory, specifically the concept of cognitive polyphasia (Moscovici, 2000).

### 1.2 Cognitive polyphasia: understanding ambivalence in perceptions

The social representation theory examines how collective cognitions are produced and transformed in societies and cultures through communication, focusing on the socio-cognitive processes or mechanisms involved (Moscovici, 1988, 2000). As such, social representations are culturally shared sets of understandings of socially prevalent realities. They are “a form of knowledge, socially produced with a practical function, namely to contribute to the construction of a reality shared by a social group or entity” (Jodelet, 1991, p. 36). In this sense, social representations capture how people make their world meaningful by observing communication processes that determine the content and structure of beliefs and practices (Moscovici, 1988). They are actively produced and developed by members of societies in de-traditionalized, fluid, and dynamic public spheres. These de-traditionalized public spheres are characterized by equality and allow us to connect to and draw on information from very different worldviews. The legitimacy of understandings is established through rational discussions where arguments of authority are being replaced by the authority of arguments (Jovchelovitch, 2007). In conclusion, social representations are not created by individuals in isolation but by discussion in societies. Once created, they “lead a life of their own, circulate, merge, attract and repel each other, and give birth to new representations, while old ones die out” (Moscovici, 1988, p. 13). Individuals and groups create, shape, and re-shape representations through communication and interaction by eliciting intra-individual “anchoring” and “objectification”’ processes (Moscovici, 1988, 2000). Anchoring refers to classifying and naming a social reality, whereby a set of rules, characteristics is ascribed to a social reality, stipulating what is permissible in relation to all members of the group and delimiting the new representation from others. Anchoring is thought to be the dominant process in the case of a representation remaining unthematized and unproblematized. Then, typical points of view and common perspectives are recycled and reformulated—the ontology of a representation is taken for granted. In comparison, objectification is assumed to be the predominant process when representations become challenged during times of crisis, societal tension, and upheaval (Moscovici, 2000; Duveen, 2001). Then, the individual is challenged to find justifications for existing beliefs and attaches important new meanings to representations (Markova, 2000).

Objectification processes are also at the root of cognitive polyphasia (Moscovici, 1988, 2000). Cognitive polyphasia, first coined by Moscovici (1988), describes that individuals may be able to hold incompatible representations that refer to the same representation but organize and interpret this representation in distinct ways. In contrast to the concept of cognitive dissonance (Festinger, 1962) that supposes an individual’s inability to hold dichotomous representations without negatively affecting the self’s equilibrium, cognitive polyphasia assumes that, as long as each representation is locally consistent, even contradictory representations can coexist side by side. Therefore, “it is in the context of different life worlds that holding on to “contradictory” representations makes sense” (Wagner et al., 2000; p. 306). The coexistence of incompatible beliefs that form contradictory representations is thought to be a normal state of affairs in communication and ordinary life. It occurs not only in societies at variance with each other but also within individuals. In this sense, different circumstances and social contexts in which specific “truths” are accepted require the individual to form also different situationally dependent responses in order to behave in functional ways (Wagner et al., 2000; Provencher, 2011). The same representation is then conceptualized in different ways, all equally valid though contextually coherent.

In accordance with cognitive polyphasia, the same individual may hold heroizing and victimizing representations of Iraq and Afghanistan veterans. For example, Iraq and Afghanistan veterans may be discussed in victimized ways when recalling the context of the deployments in Iraq and Afghanistan and understanding these as a ‘state crime’. However, the same individual may characterize veterans in heroic ways when thinking of Iraq and Afghanistan veterans’ decision to sign up for the military and defend the U.S. overseas. This would outline the ephemeral, fluidly changing nature of representations (Frilling, 2012), allowing individuals to make sense of complex social worlds when being confronted with contesting, conflicting, and contradictory information. By drawing on the theoretical underpinning of cognitive polyphasia, the present research will examine how competing and contrasting representations of the U.S. Army Iraq and Afghanistan veterans may coexist within individuals by examining the context and conditions of their emergence. In doing so, this will provide deeper insight and add interpretational value to understand descriptive information about public perceptions of veterans gathered in representative opinion polls and surveys.

## 2 Methods

### 2.1 Participants and procedures

To illuminate ambivalent perceptions of veterans in individual thinking, the present project adopted a qualitative approach. After receiving ethics approval from the University of Colorado, Colorado Springs (IRB 19-064), a sample of 29 individuals (17 male/12 female) was generated. It has previously been recommended that qualitative studies require a minimum sample size of at least 12 to reach data saturation (Guest et al., 2006; Braun and Clarke, 2013; Fugard and Potts, 2015). Therefore, a sample of 29 was deemed sufficient for the qualitative analysis and scale of this study. Participants were recruited by advertising the project at local public notice boards, through different social media channels and through word of mouth. All participants were previously unknown to the researcher, and only professional contact was maintained to receive participants’ approval of the transcribed interview after the interviews. As social contact with U.S. Army Iraq and Afghanistan veterans has been identified as a significant predictor for holding victimizing perceptions about veterans (Phillips and Albanesi, 2022), the present study recruited individuals with varying degrees of social distance to U.S. Army Iraq and Afghanistan veterans. Of this sample, 10 individuals (five male/five female) identified as not knowing U.S. Army Iraq and Afghanistan veterans, 10 individuals (four male/six female) identified as being in close social contact with U.S. Army Iraq and/or Afghanistan veterans, and nine individuals (eight male/one female) identified as U.S. Army veterans who had been deployed in these conflict zones. In addition, also differences in educational attainment were considered in the recruitment of the participants. An overview of the participants with their sociodemographic data can be found in Table 1. A semi-structured interview schedule was utilized to elicit individual perceptions of U.S. Army Iraq and Afghanistan veterans, following the interview structure of the BNIM (Wengraf, 2001). In accordance with the BNIM (Wengraf, 2001), the initial section encouraged participants to speak freely about their perceptions of U.S. Army Iraq and Afghanistan veterans (*“When you think of U.S. Army veterans who were deployed to Iraq or Afghanistan, which thoughts come to your mind?”*). This section aimed to open up a participant-guided narrative to gain original and unique understandings of the topic in question (Wengraf, 2001). This section was essential for the purpose of the present study as it provided space for the emergence of possibly dichotomous or contrasting representations of veterans as heroes and as victims. The second part consisted of the interview was based on the participants’ responses to the initial question (SS1; Wengraf, 2001). These follow-up probes (SS2) encouraged the participant to elaborate on information and to challenge contradictory perceptions of veterans to clarify the respondent’s meanings. This contributed to illuminating the contextual dependency of contrasting representations, and to understanding how contradictory representations of veterans—when challenged—may be rationalized (e.g., *“You mentioned ‘damaged’. What do you mean with[sic] that?”*). The final sub-section (SS3) comprised specific questions that were based on pre-existing knowledge, by integrating information from the reviewed literature. For this purpose, previous literature on public perceptions of veterans was accessed (*cf.* literature cited in the introduction) to ask relevant questions that clarify how individual understandings of veterans may be rationalized. These questions represented changes in personal perceptions regarding the veteran and understandings of socially prevalent perceptions and attitudes toward veterans (e.g., *“Why do you have this opinion of U.S. Army Iraq and Afghanistan veterans? Was there a time in your life when you thought differently about U.S. Army Iraq and Afghanistan veterans?”*).

**Table 1:** Overview of the participants and sociodemographic characteristics.

Social distance	Age range	Education	Sex	Identifier
No Contact	55+	Below A Levels/High school Diploma	Male	1-NC
No Contact	55+	Below A Levels/High school Diploma	Male	2-NC
No Contact	55+	Above A Levels/High school Diploma	Female	3-NC
No Contact	18–34	Above A Levels/High school Diploma	Male	4-NC
No Contact	18–34	Below A Levels/High school Diploma	Female	5-NC
No Contact	18–34	Above A Levels/High school Diploma	Male	6-NC
No Contact	18–34	Below A Levels/High school Diploma	Female	7-NC
No Contact	18–34	Above A Levels/High school Diploma	Female	8-NC
No Contact	18–34	Above A levels/High school Diploma	Female	9-NC
No Contact	18–34	Below A Levels/High school Diploma	Male	10-NC
Close Contact	55+	Below A Levels/High School diploma	Male	11-CC
Close Contact	55+	Below A Levels/High School diploma	Male	12-CC
Close Contact	55+	Above A Levels/High School diploma	Female	13-CC
Close Contact	35–54	Above A Levels/High School diploma	Female	14-CC
Close Contact	18–34	Below A Levels/High School diploma	Female	15-CC
Close Contact	18–34	Above A Levels/High School diploma	Male	16-CC
Close Contact	18–34	Above A Levels/High School diploma	Female	17-CC
Close Contact	18–34	Above A Levels/High School diploma	Female	18-CC
Close Contact	18–34	Above A Levels/High School diploma	Male	19-CC
Close Contact	18–34	Above A Levels/High School diploma	Female	20-CC
Iraq/Afghanistan veteran	55+	Below A Levels/High School diploma	Male	21-V
Iraq/Afghanistan veteran	35–54	Below A Levels/High School diploma	Male	22-V
Iraq/Afghanistan veteran	35–54	Below A Levels/High School diploma	Male	23-V
Iraq/Afghanistan veteran	35–54	Below A Levels/High School diploma	Male	24-V
Iraq/Afghanistan veteran	35–54	Below A Levels/High School diploma	Male	25-V
Iraq/Afghanistan veteran	35–54	Above A Levels/High School diploma	Male	26-V
Iraq/Afghanistan veteran	35–54	Above A Levels/High School diploma	Female	27-V
Iraq/Afghanistan veteran	18–34	Above A Levels/High School diploma	Male	28-V
Iraq/Afghanistan veteran	18–34	Above A Levels/High School diploma	Male	29-V

The interview questions were piloted and trialed before the participants were interviewed. For this purpose, three pilot interviews were conducted that were not included in the present analysis. The pilot interviews were conducted before the main data were collected and the pilot interview participants were recruited through the same means as the participants for the main study. The pilot interviews were transcribed and checked whether the questions asked provided information about the research question. Here, irrelevant SS3 question items were deleted that, while interesting, did not provide substantial information on representational construction (i.e.*, ‘When you told me about what you understand of an Iraq or Afghanistan Army veteran in question 1, you said that this person was [xxx]. Did you think of a particular gender? Why do you think you associated this particular gender?’*). After these minor amendments to the interview schedule were made in order to make the schedule more concise, the interviews for the main study were conducted. All interviews were audio-taped and anonymized during transcription. After the transcripts were sent to participants for checking, a thematic (Braun and Clarke, 2006) analysis was conducted.

### 2.2 Data analysis

A combination of an inductive-deductive thematic analysis (Braun and Clarke, 2006) allowed a thorough examination of multiple perspectives that are involved in forming, contesting, combining, and negotiating individual perceptions of U.S. Army Iraq and Afghanistan veterans and how these perceptions may relate to each other. After the data have been transcribed, initial ideas about key topics, relationships between different perceptions of veterans, and potential themes were noted, using NVivo 12. The data were reread and reviewed to identify potential key ideas that emerged repeatedly that may form themes. Here, the analysis was guided by a primary and secondary focal point. The primary focal point examined social representations of veterans. In this step, the analysis examined differences in individual understanding of meanings associated with U.S. Iraq and Afghanistan veterans by drawing upon perceived central characteristics of U.S. Iraq and Afghanistan veterans (Jovchelovitch, 2007). In this sense, the first step allowed us to define and differentiate between social representations of veterans as being victims or heroes. This was important as the semi-structured interview schedule gave participants the opportunity to elaborate on their unique and original perceptions of typical characteristics of U.S. Army Iraq or Afghanistan veterans. The second focal point of the analysis investigated the interaction of heroic and victimized representations of veterans, by examining the conditions of their emergence. To understand how contrasting, even dichotomous representations of veterans as heroes and victims could be held by the same individual, the contextual dependence of heroizing and victimizing characterizations and switch-over points between hero-victim representations were examined. The narrative accounts were then compared and contrasted to identify similarities and interpreted with cognitive polyphasia as a theoretical backdrop to explain the coexistence of victimizing and heroizing beliefs. This allowed the identification of connected thematic properties that, drawn together, helped highlight the way how different representations of U.S. Army Iraq and Afghanistan veterans were connected through contextual views and information.

## 3 Results

The results also outlined that from 29 respondents, a total of 26 participants utilized both, heroic and victimizing references in describing veterans, while one participant utilized only heroic and two participants only victimizing references in descriptions of veterans. Importantly, the results also outline that cognitive polyphasia may explain how individuals may hold victimizing and heroizing representations as these depend on contextual cues that relate to the legitimacy of the mission that veterans fought. Although individuals from different social backgrounds and varying degrees of social contact with the U.S. Armed Forces were recruited, the analysis did not indicate differences in answering patterns. In all groups, evaluations regarding the legitimacy of the missions impacted descriptions of veterans in similar ways, allowing them to understand veterans in contextually coherent ways, being both heroes and victims of warfare. Evaluations of the legitimacy of the missions (Theme 1: Individual Understandings of the Deployments to Iraq and Afghanistan) impacted understandings of the battlefield (Theme 2: Conceptualizations of war, deployment, and violence) and the psychological damage associated with veterans due to attributions of morally superior personality dispositions (Theme 3: Evaluations of the veteran’s personality). In conclusion, the following results outline that individuals may hold dichotomous representations of veterans that, while being globally inconsistent, make sense to the individual as being contextually consistent.

### 3.1 Theme 1: Individual understandings of the deployments to Iraq and Afghanistan

Whether participants characterized veterans in heroic or victimized ways strongly depended on their understanding of the deployments’ legitimacy. However, from the 29 participants, a total of 26 held oppositional and conflicting understandings of the deployments. In some contexts, the deployments were considered to be justified; in others, the deployments were perceived to be illegitimate. Participant 5-NC explains:

“Well, you see, for me—I wasn’t there. It’s difficult to tell whether it was ok to send out troops there. Ok, we did not find weapons of mass destruction. But I even cannot say that for sure. There is so much on social media – some say this, others say that. When it comes to weapons of mass destruction, I’d say the missions were not justified. Did the government send them to secure oil money? If so, they’d not be legitimate.

[…]

I’d definitely say we have accomplished our mission goals. We’ve tried to teach them how democracy works, for example… And women’s rights. And that were concepts, these people have not heard about before.”

Similarly, 22-V states:

“Nah, I do not think we should’ve gone there in the first place. We had no place to be there, I think… I think the government set us up—cause war is good for the economy, y’know?

[…]

We did fight terrorism—did fight these damn terrorists… and somebody had to do it. If it wasn’t us—who else should’ve done it? Cause it kinda was important… so you see—we had to go and just… do … do something about these terrorists before they attack us in our front gardens….”

Both of these quotes highlight a tendency evident across 26 of the 29 interviews. Participants were generally unsure about the mission objectives for the Iraq and Afghanistan deployments. With obscured and diffuse mission goals and objectives, such as finding weapons of mass destruction but also labeling the missions in Iraq and Afghanistan as counter-insurgency campaigns, individuals utilized contesting rationalities to justify why the missions were legitimate or illegitimate. Essentially, these quotes demonstrate how participants may have competing and contesting representations of the Iraq and Afghanistan wars—being legitimate and illegitimate deployments. Different circumstances and social contexts require the individual to form different situationally dependent responses to behave in functional ways (Provencher, 2011). The participants’ knowledge of the missions in Iraq and Afghanistan is created by drawing on public dimensions that do not provide the individual with a consensus of opinions, a typical characteristic of social realities (Jovchelovitch, 2007). Instead, the participants are placed within a field of tension that is created by contesting and equivocal information which individuals need to assess and examine to form personal beliefs about a social reality. In the present example, participants reiterated contrasting views drawn from public spheres (e.g., “there is so much on social media” 5-NC, “people say that it was about oil money” 17-CC), found arguments, although opposing, equally valid, and so situated the arguments within different contexts to perceive contextual consistency. In this sense, the participants were able to see the missions in Iraq and Afghanistan as being legitimate and illegitimate missions—depending on the framing of the missions. However, when the interviewer confronted participants with dichotomous statements regarding the legitimacy of the missions, participants found a way to combine contrasting arguments by forming an overarching rationality. This rationality contributed to situational coherence by providing a hierarchical structure, which allowed to order the relevance of—even dichotomous—arguments. In this way, participants developed a ‘favored framework’, when prompted, that they perceived to be most aligned with their personal values. For example, participant 11-CC suggests:

“[…] So it’s a yes and a no – they are both [legitimate and illegitimate]—depending on the way you look at it. […] … and so for me, it’s tricky.[…] But at the moment, I am just unhappy with our government. They got so many things wrong…. And Iraq and Afghanistan… that was one of the them. They did not check the intelligence… they made a lot of mistakes… and they sent them [Army] off to the unknown. I do not believe that we have achieved a lot there, so maybe the negatives outweigh the positives. Maybe it was all because of money and economy.”

Participant 23-V:

“You can always argue for and against a mission – it’s never clear cut. We went there to do one thing and got something else out of it… cause maybe our intelligence was flawed– but we did a lot, not all was bad. I think, overall, we have had a positive impact, a very positive one. We have helped so many civilians over there. And that’s what it means to be a veteran… that’s why I joined, I wanted to help others.”

Both of these accounts outline how participants were able to find an ultimate “truth” while considering different competing and opposing opinions as equally true. Importantly, while being able to hold contesting views, one opinion was considered “truer” than another if nudged toward different contexts.

### 3.2 Theme 2: Conceptualizations of war, deployment, and violence

Nested within contextually dependent frameworks of the legitimacy of the missions were conceptualizations of war, deployment, and violence, and how these may impact the veteran who has witnessed war, deployment, and violence. While all participants characterized deployment as an unhealthy environment that would inevitably contribute to the development of mental health problems, the legitimacy of the deployments was crucial in understanding long-term effects. While the mental health problems that arose from “good” or legitimate violence were considered to be treatable, serious psychological impairments were attributed to ‘bad’ or illegitimate violence. Here, different forms of guilt (e.g., guilt due to violation of the own ethical conduct and survivor guilt) were particularly important in forming an underlying rationale that justified anticipations of mental health problems. However, which form of guilt was anticipated and perceptions of how deeply guilt would affect the veteran depended on the evaluation of the deployments’ legitimacy. If the deployments in Iraq and Afghanistan were understood as being justified, then veterans were considered to suffer from witnessing atrocities and committing violent actions in ways that would diminish over time and can be treated. For example, 18-CC stated as follows:

“They [veterans] were brave for executing this deployment goal—the terrorists had to be eliminated. And yes, of course, they will have some problems—PTSD, TBI, depression. But I do not think… I do not think this would affect their mental health in the long run. It was for a purpose—it was necessary to make these countries safer.”

Similarly, 26-V states as follows:

“Negatives [of deployment] being TBI, PTSD, anxiety—things I have not had before. But I have been taking medication for it. And it helps me when I think about the positive contribution that I have made. Overall, I feel that it was still a positive experience. And I think the problems I have now will go away because I get all the help I need.”

These quotes outline how anticipations of a purpose inform understanding of the severity of mental health conditions. The accounts indicate purpose to be a crucial element in the evaluation of the veteran’s capability to overcome mental health problems. However, if violence was committed in illegitimate conceptualizations of deployments, then veterans were considered to suffer from lifelong psychopathological conditions. For example, V-28 states as follows:

“I guess it has to be very difficult to know that you have committed … what can be considered as a crime. You’d probably be quite troubled in that sort of scenario. And PTSD, nightmares, anxiety—it’s gonna affect you… it’s gonna affect you, I think.”

Psychological impairments become therefore a “termed personification” (Joffe, 2007, p. 206), a graspable reality that substitutes the abstract idea of punishment for illegitimate actions. In this way, observable negative consequences of amoral behavior visualized both, social ideals of altruism and the violation of these ideals, by transforming them into concrete realities. Importantly, psychological impairments as punishment for unjustified actions were situational dependent and could only be observed in the context of illegitimacy. Veterans then became the victims of their experience. For example, participant 14-CC explains as follows:

“I think killing is something you would never forget. Especially in a war without purpose. You know, it took a lot of time, he [spouse who is a veteran] refused to go to counsellors, he did not take drugs or alcohol, but it took him more than 10 years to get over it. […] I do not know but I think the experience for him might have been different if he knew why he had to do it. What the purpose of taking this life was—was he a terrorist?”

This quote demonstrates how conferring own perceptions of the deployments’ legitimacy justified associating the experience of shame and guilt with veterans who returned from Iraq and Afghanistan. Here, the attribution of experiencing guilt allowed the participants to maintain a positive perception of veterans, while disregarding morally reprehensible acts. However, although veterans were considered to experience guilt, the participants dissociated veterans from culpability. Instead, the government or unfaithful superiors were blamed for what was perceived as being an illegitimate mission or unjustified killing. This elicited pitiful and compassionate responses as the following example from 19-CC shows as follows:

“They go there [Iraq/Afghanistan] for no reason, for no reason, for what? There is no personal gain for you! They have to follow their orders and […] they are not themselves anymore when they come back. And there is no help for them coming back, they have all sorts of problems and nobody cares. It’s just really sad.”

In this sense, participants took a moral and ethical perspective in evaluating the veteran’s guilt (Gamlund, 2013). On the one side, the veteran was understood as having done wrong through the involvement in immoral acts. On the other side, the veteran was perceived as having a justification (i.e., acted to the best of his knowledge at that time and lawful self-defense) or an excuse (following the orders, no possibility to be disobedient) for their actions. The veteran’s participation in violence was thus excused as “to regard a conduct as excused is to admit that the conduct was wrong but to claim that the person who engaged in the conduct lacked substantial capacity to conform his conduct to the relevant norms and thus was not fully a responsible agent.” (Gamlund, 2013, p.109). This allowed us to attribute low fault, blameworthiness, and culpability to the veteran. Participant 1-NC explains as follows:

“They were made to go there, did not accomplish anything … but the veterans, they were forced to harm people. And that’s what they got out of it. You signed your life away cause you have trusted in the state and they cheated you. And the state decides to do stupid things with you and you… you cannot just opt out. You go there for no reason and come back with all sorts of issues. And the state does not take on any responsibility for what they did to you.”

This quote exemplifies how the U.S. government was described anthropomorphically, characterized by human traits such as dishonesty and betrayal. This allowed them to continue to hold Iraq and Afghanistan veterans in high regard even when perceptions of veterans were discussed in the context of illegitimate warfare. As a result, unconditional support for Iraq and Afghanistan veterans was evident in all accounts, or in the words of participant 17-CC “even if you do not support the war, support our veterans.” Similarly, participant 8-NC suggested: “We need to support U.S. veterans, no matter what they did over there [Iraq/Afghanistan].”

### 3.3 Theme 3: Evaluation of the veteran’s personality and dispositions

To understand the Iraq and Afghanistan veterans’ voluntary decision to serve the country, the participants associated specific personality traits with veterans. Essentially, conceptualizations of war and deployment as being necessarily damaging justified the rationale that only a specific group of people would join the military. This specific group of people was thought to be inherently different from civilians, motivated by superior morality and altruism. In the context of the legitimized deployments, the veteran was conceptualized as a protector of specific threats (i.e., terrorists) that endanger societal peace and harmony. Essentially, the abstract notion of a morally superior personality was conceptualized through “bravery”—the willingness to experience existential threats to other people’s wellbeing. Therefore, altruistic deeds, heroism, and bravery stood in a reciprocal triad, each being used to define the other, as participant 10-NC explains:

“The fact that they were willing to put down their own life to protect others makes them brave. And that they were willing to stand up in the most dangerous way possible […] to make sure that other people would not have to do that. That’s very heroic.”

As these quotes outline, understanding of the veteran’s personality was highly intertwined with bravery. Bravery became therefore the “ultimate” word to describe the veteran. Crucially, bravery was thought to be a natural disposition, an expression of a more altruistic personality. In this sense, the veteran was thought to have an inherently different personality. This personality was characterized by supreme moral values that the veteran was willing to defend. Participant 8-NC elaborates as follows:

“I guess it takes a certain kind of person. Like I said before, you need to have some level of bravery, I imagine they are pretty … maybe it’s not something you develop… you are just born like that, right? It’s just… it’s just nothing I’d be able to do.”

This quote outlines how understanding of bravery as related to a specific, inherently different personality transformed the meaning of what it means to be a veteran. ‘Veteran’ therefore turned into an expression of the individual rather than seeing the individual as a veteran. In other words, to be a veteran was considered a destiny. The understanding that heroic deeds could only be done by heroic people was constructed, therefore the social category ‘veteran’ reflected also self-positioning toward service. This allowed the non-veteran population to distance itself from service. In conclusion, non-veterans maintained a positive identity structure by understanding themselves as having a different nature and thus a different place in society. As participant 17-CC describes: “My brother has always… he’s just been different, right?.” Or as participant 19-CC explains: “We all have different duties. I have mine and the veteran has his.”

Descriptions of altruistically superior personality characteristics as typical characteristics for veterans were reiterated by veteran participants. Although an ideal type and supreme veteran personality was described, having this personality oneself was frequently rejected. Instead, the brave personality was thought to be the reason why other veterans had joined, while understanding one’s own motivation to serve as being a family tradition, having more self-serving origins (i.e., adventure, social contacts, and opportunities), or a combination of both. Participant 22-V explains:

“I mean for me, I was shit at school at that time so joining the military was a step towards having a purpose. But as a child, I never thought about joining. It was later […] but knowing that my dad had served made it easier. But people have different reasons, most of them are just good people who want to help others.”

This quote illustrates the often-contradictory nature of the veteran’s self-positioning toward the understanding of veterans as heroes. Veterans were often aware that their own personality might not be as altruistic/brave as the socially prescribed veteran personality. However, lauding veterans for the highest moral values that originated in an altruistic disposition positively impacted the non-veteran’s and the veteran’s self-image. On the one side, it allowed non-veterans to reflect on having a different place in society and appreciate the sacrifices that they did not have to make. On the other side, veterans felt appreciated by social attributions of a specific character that made the veteran inherently different from individuals in civilian society.

However, in the context of the illegitimate mission, attributions of moral superiority backfired as these were associated with an increased likeliness of suffering from mental health problems as violating the anticipated moral conduct of veterans. Participant 19-CC explains:

“You want to be a hero, sign up, cause you think this will give you the possibility to do all the right things… help people who need help… and then… it’s sad to know that they have to do things that are so horrific… awful. Someone who has this sense to help others, and he has to take the lives of innocent people… How is he gonna live with his conscience? I guess he will take whatever substance he can get his hands on when he returns home … you know, just to cope with these experiences—because he never intended to do harm anyone… how is he going to handle it? My guess—not well.”

As this quote demonstrates, mental health illnesses are not only anticipated but also considered life-wrecking necessities following the experience of illegitimate violence. In this sense, veterans are associated with becoming “innocently” involved in unjust violence, ending up “unintentionally” on an illegitimate battlefield. Associating well-intending unknowingness to the veteran’s personality allowed the creation of a symbolic distance between the veteran and the war, facilitating holding the veteran in positive regard, while simultaneously opposing the deployments in Iraq and Afghanistan. However, because of the morally superior personality, injustice and illegitimacy were considered a life-wrecking experience. In the words of the participant 17-CC, “They have this special sense for duty, for helping those in need. And then, they are forced to do the opposite—to harm those they wanted to help. How could they possibly live with themselves, knowing what they did. Others might be able to—but not a veteran.”

## 4 Discussion

The present study represents a qualitative approach to understanding the veteran hero-victim relationship. The results suggest that the understanding of veterans may directly relate to the missions they fought. However, understanding of veterans, which is embedded within situational and contextual information surrounding the deployments they returned from, is as ephemeral and fluidly changing as the individuals’ perceptions of the deployments. This may be explained by individual attempts to integrate complex and dichotomous arguments surrounding war and deployment in Iraq and Afghanistan, prevailing in de-traditionalized, fluid, and dynamic public spheres. Although anchoring and objectification processes allow the integration of competing, contextually dependent arguments, to develop a fully fledged understanding of war and deployment to Iraq and Afghanistan, these understandings impact the emergence of social representations of veterans who returned from these missions and are held by the individual. In this sense, social representations of veterans as both, victims and heroes, can reside in the same individual as their emergence depends on contextual cues, both to be perceived equally—or almost equally—legitimate by the individual. Social representations of veterans as ephemeral and fluidly changing represent therefore the very nature of the conditions under which social representations evolve: dynamic communication processes that determine the content and structure of beliefs and practices (Moscovici, 1988). Even if objectively contradictory or incompatible, social representations of veterans as both, victims and heroes, make sense to the individual, as responding to different circumstances and contexts that require specific “truths” to be accepted in order for the individual to behave in functional ways (Wagner et al., 2000; Provencher, 2011). Therefore, in the context of complex social worlds, where competing and contesting information is presented, the coexistence of what seems to be objectively incompatible beliefs becomes a normal state of affairs in communication and ordinary life. In conclusion, the concept of cognitive polyphasia (Moscovici, 2000) may indeed explain ambivalent results on how the U.S. public may perceive veterans outlined in numerous opinion polls, surveys, and empirical studies (Pew Research Center, 2011, 2018, 2019; Greenberg Quinlan Rosner Research, 2012; Veterans and Citizens Initiative, 2022; Wenig et al., 2023). Positive and supportive attitudes toward veterans may not solely represent socially desirable reporting as earlier studies had suggested (e.g., Kleykamp and Hipes, 2015). Instead, holding appreciative attitudes while also holding victimizing sentiments toward veterans may be the result of the individual sense making processes of complex and contesting information, widely available in the de-traditionalized public spheres, in which the authority of arguments overruled the arguments of authority (Jovchelovitch, 2007; Armstrong and Olatunji, 2009; Bragin, 2010; Kleykamp and Hipes, 2015; Rhidenour et al., 2019).

However, in addition to outlining that the veteran hero-victim relationship may be explained by two realities residing in different contexts, the present project also examined the context in which the different representations reside in greater detail. In doing so, this is the first study that provides empirical evidence, outlining the impact of societal framing of deployments and mission objectives on social representations of veterans. Essentially, the veteran victim representation may be culturally prevalent, not only because it resides in popular media but also because it may be based on the veteran-hero representation. When forced to make sense of conflicting information regarding mission goals, objectives, and success, the U.S. government is utilized as a scapegoat to avoid culpability and blame in relation to veterans and the U.S. Military. While the present study therefore contends with previous research suggesting that the majority of the U.S. may differentiate between their perception of missions and veterans who returned from these missions (Pew Research Center, 2011, 2018), the present results also outline that the public may do so by focusing on anthropomorphic descriptions of a deceitful U.S. government. In this sense, veterans are conceptualized in a victimological context: obliged by their military contract to violate human rights standards, turning them victims of a ‘state crime’ (Kauzlarich et al., 2001; Ruggiero, 2005; McGarry, 2012). Heroizing and victimizing representations of veterans may therefore be built upon the same underlying sentiments and rationalities: on the one side, veterans are supposed to have morally superior and dedicated personalities, and, on the other side, deployment equates with witnessing horrific and amoral violence. However, the perceived legitimacy of the deployments is the decisive factor that explains whether the necessarily incurred deployment-related mental health problems are short-lived and curable or long-lived and life-wrecking.

In conclusion, anticipations of veterans as damaged, labile, and fragile individuals outlined by previous research (Hoge et al., 2006; Pew Research Center, 2011, 2019; Hipes et al., 2015; Schreger and Kimble, 2017) may indeed pose a significant risk for an adequate reintegration of veterans, even if held implicitly. However, as the veteran-victim representation was found to be highly intertwined with the veteran-hero representation, it may be difficult to dissociate veterans from implicit and explicit stereotyping that relates to victimizing sentiments (Stone et al., 2018; Hipes and Gemoets, 2019). The present results would suggest two options that may allow to dissociate veterans from stereotypical victimizing sentiments. First, a dissociation between veterans and victimizing sentiments may be achieved by engaging in a sensitive assessment and reflection on mission goals and objectives, to understand whether these are backed by the general public. Mission goals in relation to the deployments to Iraq and Afghanistan were perceived to be fluid and changing. This uncertainty about what veterans ought to achieve in Iraq and Afghanistan contributed to the victimization of veterans. Therefore, unequivocal mission goals and objectives need to be presented to the public at the onset of any mission to avoid the victimization of veterans. Alternatively, it may be helpful to dissociate veterans from publicly pertinent victimizing sentiments by addressing the superiority of a veteran’s personality and providing fact-based information about deployments. Anticipations of psychopathologies are considered to be caused by moral injury (*cf.* Shay, 2014; Richardson et al., 2020; Wilson et al., 2024), undermining the veteran’s moral codex. As such, both, anticipations of altruistically superior personalities and illusionary battlefields, based on representations of battlefields in today’s media, contribute to associating victimizing sentiments with veterans. As such, it may be helpful to provide fact-based information about the deployments in Iraq and Afghanistan, focusing on technological progress and the variety of roles that veterans of these deployments held. In addition, promoting understanding of being a veteran as an end state of a—special—occupation may allow to dissociate veterans from anticipations of altruistically superior personalities and therefore make attributions of ill health less likely. In this way, public perceptions of needy veterans who returned from nasty deployments may be challenged and reframed, contributing to a non-stereotyped reintegration of Iraq and Afghanistan veterans in U.S. civilian society.

While the present study provided a cohesive examination of the veteran hero-victim relationship, a number of caveats need to be taken into consideration. One limitation addresses the choosing of branches of military personnel. The present study focused on Iraq and Afghanistan veterans who were part of the U.S. Army. It may be possible that different rationalities are utilized for veterans from other branches. For example, it may be possible that veterans from the U.S. Air Force are perceived differently, as being less associated with front-line fighting. Similarly, people may hold different rationalities, justifying the victimization of veterans from the U.S. Navy. Similarly, the present study focuses on veterans who returned from specific deployments to Iraq and Afghanistan only. Therefore, caveats regarding the present findings’ transferability need to be taken into consideration. Future research may want to address this gap by examining whether the extent of public victimization of veterans from different branches and different deployments significantly differs, and, by examining rationalities leading to the victimization of veterans that may be unique to different branches or different deployments. Another limitation concerns the analytic options chosen in the present study. Essentially, constructing the themes that evolved from answering patterns contributed to overlooking more implicit themes. This may be particularly the case for the present study as the focus was placed on victimizing and heroizing sentiments in characterizations of veterans. For example, notions of veterans as ex-employees or perpetrators, that were sparsely mentioned in the narrative accounts, may not have received sufficient attention. In addition, political affiliation may be an important factor in individual perceptions and characterizations of veterans. This may be the case as previous studies suggested a relationship between political affiliation and perceptions of joining motives of U.S. veterans (Krebs and Ralston, 2020). Specifically, conservatives idealized the image of service members as moved by self-sacrificing patriotism, and liberal Americans considered economic reasons as driving motives into service. Therefore, victimizing sentiments may be more prevalent in liberal cohorts who think that veterans have joined mainly to escape desperate circumstances. However, while interesting, this was not the main intention of the present study. Although previous studies have outlined that party affiliation, together with other sociodemographic characteristics, may impact perceptions of veterans, it is not granted that individuals think of veterans as only heroes or only victims. In examining the ephemeral nature of the veteran hero-victim relationship instead of examining the impact of sociodemographic characteristics, the present study followed its main objective. Finally, the small sample size of 29 individuals may be subject to scrutiny. Although a heterogeneous sample was sought with participants varying substantially in sociodemographic backgrounds and their relationship to the U.S. Military, a relatively small sample size of 29 participants may not allow to make substantial conclusions about the relationship between heroizing and victimizing representations of veterans who returned from the U.S. deployments to Iraq and Afghanistan. However, as previous research focusing on identifying appropriate sample sizes for thematic analysis deemed 12 interviews to be sufficient (Guest et al., 2006; Braun and Clarke, 2013; Fugard and Potts, 2015), a total sample of 29 interviews was considered to be adequate to analyze answering patterns in relation to representational construction of U.S. Army Iraq and Afghanistan veterans.

In conclusion, the present study suggests that perceptions of U.S. Iraq and Afghanistan veterans are nested within individual perceptions of the missions that these veterans fought. Importantly, individuals may hold different perceptions of the missions that veterans returned from, which is why also different perceptions of veterans who were deployed to these missions can be held. As such, the present study found cognitive polyphasia a useful theoretical foundation to examine societally prevalent representations of veterans and interactions of these representations in depth. The results suggest that dissociating veterans from victimizing sentiments may be a difficult undertaking. This is particularly the case for ambivalently perceived wars, characterized by dubious or scrutinized mission objectives and unclear markers of victory, as these may represent a foundational element in the making of social representations of victimized veterans. Ambivalent wars may provide varying arguments that contribute to rationalities justifying victimizing perceptions of veterans (e.g., victims of the battlefields with physical or mental health problems, victims of a society that does not provide sufficient aid, and victims of a government that deployed veterans imprudently or for selfish gains). Narratives surrounding the suffering veteran are then readily available in the public conscience and reproduced in social representations of veterans who returned from specific wars. To improve public perceptions of veterans, it may be beneficial to change political, social, and media narratives of the Iraq and Afghanistan wars. While difficult in hindsight, compelling and unified narratives surrounding reasons for the deployment may improve perceptions of veterans and may dissociate veterans from harmful pitying and victimizing representations of veterans. In this sense, it is essential rigid assessments of deployment goals and a thorough assessment of public opinions toward potential deployments are an essential pre-deployment undertaking. In addition, analyses of public opinions of international deployments suggest that the longer a war drags on, the more likely it is to lose public support (Hallin, 1989; Pew Research Center, 2007; Gartner, 2008; Jones, 2010), it is essential to keep any deployment as brief as possible. In addition, the results may suggest that attributions of specific veteran personalities proved to be problematic. Essentially, heroic representations of veterans were based upon the notion of superior moral traits, inherent to veterans. The motifs of veterans joining the U.S. Military related to anticipated identities resembling motivated citizen soldiers, inspired by moral supremacy, inherent to personality. While these anticipations justified lauding veterans for their superior altruism and contributed to heroizing representations, an absence of legitimate mission goals and objectives undermined the veterans’ morality and so contributed to victimizing representations. Therefore, it may be helpful to present veterans as everyday people without specific character traits. Thus, existing ‘myths’ concerning the veteran’s personality may need to be challenged, and uniform mission goals that were achieved presented in order to dissociate veterans from victimizing sentiments.

## Data Availability

The original contributions presented in the study are included in the article/supplementary material, further inquiries can be directed to the corresponding author.
